# Effects of nickel sulphate and lead acetate trihydrate on heavy metal stress-related gene activities in forage pea (*Pisum sativum* ssp. *arvense* L.) in Türkiye

**DOI:** 10.3389/fpls.2025.1549488

**Published:** 2025-04-28

**Authors:** Seda Mesci, Muhammed İkbal Çatal

**Affiliations:** ^1^ Project Coordination and Guidance Office, Rectorate, Hitit University, Çorum, Türkiye; ^2^ Department of Field Crops, Recep Tayyip Erdoğan University, Faculty of Agriculture, Rize, Türkiye

**Keywords:** *Pisum sativum* ssp. *arvense*, forage pea, heavy metal stress, APX, CAT, MT, PCS

## Abstract

Researching heavy metal stress in plants is of paramount importance due to the increasing prevalence of heavy metal contamination in the environment, which poses significant risks to both plant, animal, and human health. Limited data are available on heavy metal stress-related gene responses to pollutants such as nickel sulphate and lead acetate in forage peas (*Pisum sativum* ssp. *arvense*). This study aimed to investigate how specific stress-related genes respond to stress factors such as nickel sulphate and lead acetate in this plant species. In our study, we treated three cultivars of *Pisum sativum ssp. arvense* with nickel sulfate (20 and 40 mg/L) and lead acetate trihydrate (20 and 40 mg/L). We then measured the expression of heavy metal stress-related genes (APX, CAT, MT, PCS) using qRT-PCR on three pea cultivars (Kurtbey, Kirazlı, and Pembe) in Rize, Türkiye. Down-regulations in high heavy metal treatments and heavy metal gene-associated stress tolerance expressions were detected. Additionally, high up-regulations in APX, CAT, MT and PCS gene expressions were detected mostly at high nickel sulphate and lead acetate trihydrate applied rates. The study presents up-to-date contributions to biochemical and molecular data on the effects of nickel sulfate and lead acetate trihydrate toxicity on pea plants. These insights may inform strategies to breed or produce more heavy metal resistant crop varieties.

## 1 Introduction

*Pisum sativum* ssp. *arvense*, commonly known as forage pea, is a valuable legume widely cultivated in temperate regions. Its agronomic significance stems from its adaptability, rapid growth cycle, and contribution to soil fertility (Acikgoz, 2001; Avcioglu et al., 2009). Primarily utilized for fodder, grazing, and green manure, forage pea provides essential nutrients for livestock and enhances soil structure through its efficient nitrogen fixation and rapid decomposition (Acar and Ayan, 2012). The high crude protein content of its herbage, reaching up to 20% in optimal conditions, underscores its nutritional importance (Acikgoz, 2013). In agricultural systems, forage pea is often integrated into crop rotations, serving as a preceding crop that enriches the soil, and in coastal areas, it is frequently intercropped with cereals, offering short-term grazing pastures.

The increasing prevalence of heavy metal contamination in agricultural soils poses a significant threat to both environmental and human health (Sharma et al., 2023). Anthropogenic activities, such as industrial emissions and improper waste disposal, have led to the accumulation of heavy metals in the soil, which can be absorbed by plants and subsequently enter the food chain (Jamil et al., 2024). Understanding the mechanisms by which plants tolerate or accumulate heavy metals is crucial for developing sustainable agricultural practices. Plants respond to heavy metal stress through various physiological and biochemical mechanisms, including the production of reactive oxygen species (ROS), which can induce oxidative stress and cellular damage (Soumya et al., 2022). Identifying molecular markers associated with heavy metal tolerance is essential for breeding programs aimed at developing resistant cultivars (Priya et al., 2023).

Despite the agronomic importance of *Pisum sativum ssp. arvense* and the growing concern over heavy metal contamination, there is a notable gap in the current research regarding the molecular responses of this species to nickel and lead stress. Specifically, the expression patterns of heavy metal stress-related genes, such as APX, CAT, MT, and PCS, in forage pea under varying concentrations of these metals remain largely unexplored. Furthermore, the potential of *Pisum sativum* ssp. *arvense* for phytoremediation, due to its ability to accumulate heavy metals, warrants further investigation. This study aims to address these gaps by investigating the effects of nickel sulfate and lead acetate trihydrate on the expression levels of APX, CAT, MT, and PCS genes in *Pisum sativum* ssp. *arvense* using qRT-PCR. The specific objectives of this research are to: (1) determine the expression levels of APX, CAT, MT, and PCS genes in forage pea under different concentrations of nickel sulfate and lead acetate trihydrate; (2) evaluate the molecular mechanisms underlying heavy metal tolerance in this species; and (3) provide insights into the potential of *Pisum sativum ssp. arvense* for phytoremediation. By elucidating the molecular responses of forage pea to heavy metal stress, this study will contribute to the development of strategies for enhancing heavy metal tolerance in plants, thereby promoting sustainable agriculture in contaminated environments.

## 2 Materials and methods

### 2.1 Plant material and design in forage pea (*Pisum sativum* ssp. *arvense*)

A pot experiment was conducted in a greenhouse located in Pazar district, Rize province, Türkiye in 2024. Three pea cultivars, Kurtbey (Trakya Agricultural Research Institute), Pembe incisi (Aegean Agricultural Research Institute), and Kirazlı (Mutlu Tohum, commercial company), were sown in pots containing peat. Four weeks after sowing, plants were treated with two heavy metals, nickel sulfate and lead acetate trihydrate, at concentrations of 20 and 40 mg/L. Heavy metal applications were made without mixing the soil by giving equal amounts of solution to each pot. The research was conducted according to the split-plot experimental design with three replications. Pots were placed randomly within blocks to avoid positional bias in the greenhouse. Treatments consisted of three cultivars (Kurtbey, Pembe incisi, Kirazlı) as main plots and heavy metal applications (Control, Nickel sulfate [20 mg/L], Nickel sulfate [40 mg/L], Lead acetate trihydrate [20 mg/L], Lead acetate trihydrate [40 mg/L]) as subplots. Heavy metal doses of 20-40 mg/L, which are semi-lethal levels for pea seedlings, were used to obtain similar results to the stress effects observed in aquatic plants in previous studies (Yalçın and Leblebici, 2014). Prior to harvest, morphological characteristics including plant height (cm), stem diameter (mm), node number (nodes/plant), internode length (mm), stipule width (mm), and stipule length (mm) were measured. In the greenhouse, temperature was controlled at 25 ± 2°C, humidity at 60 ± 5% and light was controlled at 16/8 hours (light/dark). Before statistical analysis, normality of the data was checked by Shapiro-Wilk test and homogeneity of variances was checked by Levene’s test. After it was determined that the data were normally distributed and the variances were homogeneous, the data were analysed using analysis of variance (ANOVA) in IBM SPSS (29.0.0) statistical software. Means were separated by Tukey’s HSD test at P<0.05 level.

### 2.2 Biological activity

#### 2.2.1 RNA isolation in forage pea (*Pisum sativum* ssp. *arvense*)

300 mg of plant tissue sample (1-15) forage pea *(Pisum sativum* ssp. *arvense)* was cut and ground in a porcelain mortar containing liquid nitrogen. 500 µL lysis buffer was added to the ground tissue and homogenized. The tissue sample was transferred to a 1.5 mL microcentrifuge tube, vortexed and incubated at room temperature for 5 minutes. 100 μL of chloroform was added to the lysate and incubated for 3 minutes at room temperature. The tube was centrifuged at 12000 rpm for 15 min at 4°C. The upper phase was transferred to a new 1.5 mL microcentrifuge tube, 250 µL isopropanol was added and mixed. By placing the column in a collection tube, 700 µL of sample was transferred to the column and centrifuged for 30 seconds at room temperature. 400 µL of wash buffer I was added to the column and centrifuged for 30 seconds at room temperature. Add 500 µL of wash buffer II to the column. Centrifuged for 30 s at maximum speed in a tabletop microcentrifuge at room temperature. 200 µL wash buffer II was added to the column again and centrifuged for 2 minutes. The column was transferred to a clean 1.5 mL microcentrifuge tube, 50µL of elution buffer was added and incubated for 1 minute at room temperature. Plant RNA was centrifuged for 30 s at RT and stocked at -20°C (Ecotech, Plant Total RNA Kit). RNA isolation, cDNA synthesis and qRT-PCR analyses of samples (1–15) were studied in single repeats.

#### 2.2.2 cDNA synthesis in forage pea (*Pisum sativum* ssp. *arvense*)

Conventional PCR (Thermo Scientific) was applied for 100 minutes at 42°C and 5 minutes at 85°C with the protocol containing 10 μL Total RNA (100ng), 4 μL cDNA Synthesis Kit, and 6 μL ddH_2_O content and amount. The synthesized cDNA products were stored at -20°C for the qPCR reaction (Ecotech, 5x First Strand cDNA Synthesis Kit).

cDNAs obtained from forage pea *(Pisum sativum* ssp*. arvense)* samples were analysed with Ecotech SYBR Green qPCR Assays kit protocol and Real Time PCR (Roche Lightcycler 96) device. Gene sequences were determined through the NCBI database. Primers required for RT-PCR were designed with the help of the NCBI primer blast program (https://www.ncbi.nlm.nih.gov/tools/primer-blast).

mRNA expression levels of heavy metal stress genes (APX, CAT, MT, and PCS) were investigated by qRT-PCR method. β-Actin was used as a housekeeping control gene. Forward and reverse sequences of the primers belonging to forage pea *(Pisum sativum* ssp. *arvense)* genes are presented in **Table 1**.

**Table 1:** Forward and reverse sequences of the primers belonging to *Pisum sativum* genes.

Genes	Forward sequences (5’-3’)	Reverse sequences (5’-3’)	TM (°C)
β-Actin	CTTCGCGGGCGACGAT	CACATAGGAATCCTTCTGACCCAT	59
APX	TGGCACTCTGGGTACTTT	GATTTGAGGGACCATGGACT	53
CAT	CTATTGGAAGATTATCATCT	AGAATTCTTGATTTCTTCTA	47
MT	ACCATCCTCAGAAGCAGCAC	TGCAAATGCAACAAGAGGTC	56
PCS	TGCACCCATCTACTGCCGAT	TCGCAACTTCAACACGCAGC	59

#### 2.2.3 qRT-PCR analysis of heavy metal stress activities in forage pea (*Pisum sativum* ssp. *arvense*)

**Figure 1:**
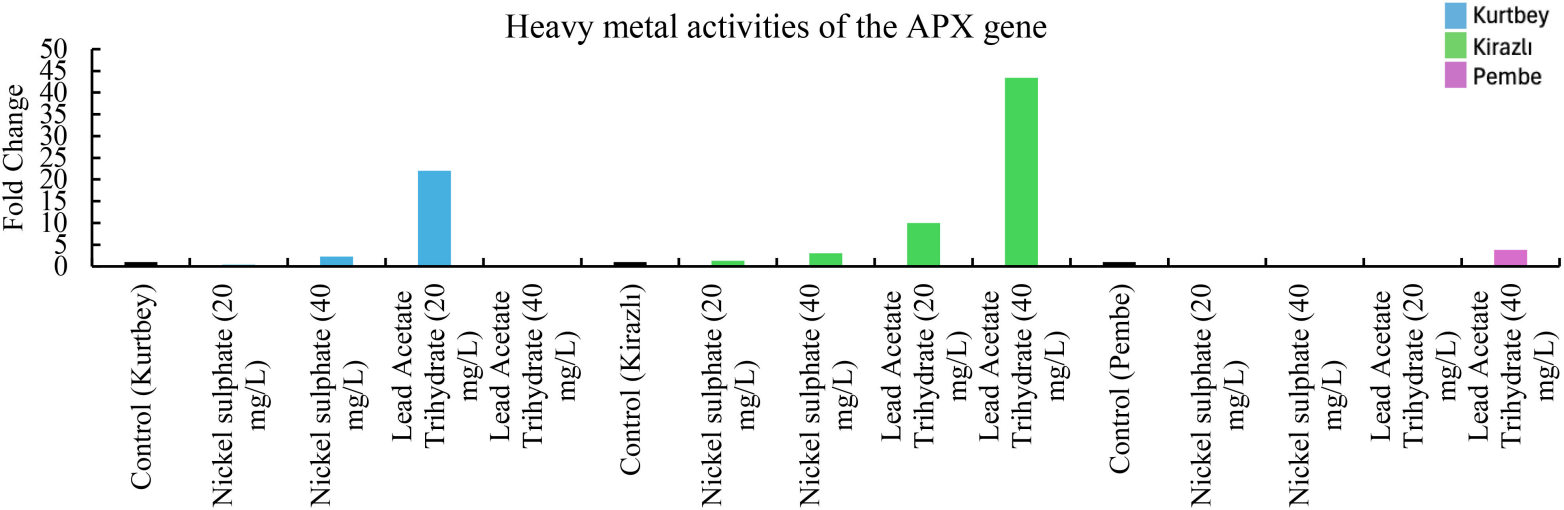
qRT-PCR mRNA gene expression levels of the APX heavy metal stress gene in *Pisum sativum* ssp. *arvesne* samples (1-15), Control: non-stressed.

**Figure 2:**
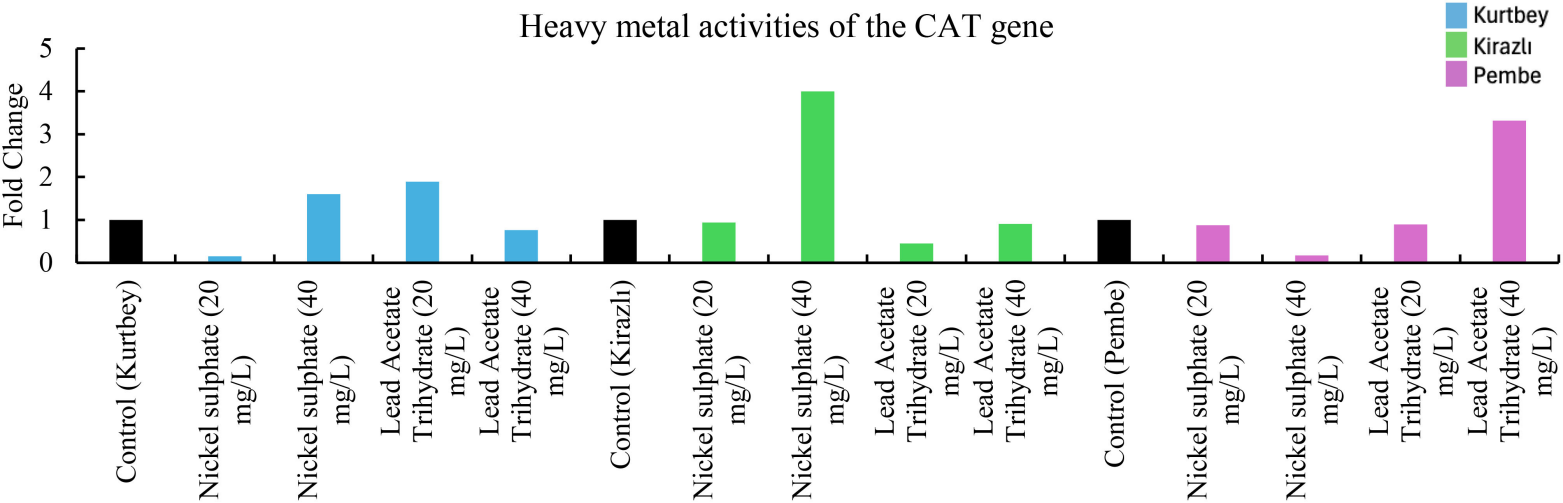
qRT-PCR mRNA gene expression levels of the CAT heavy metal stress gene in *Pisum sativum* ssp. *arvense* samples (1-15), Control: non-stressed.

**Figure 3:**
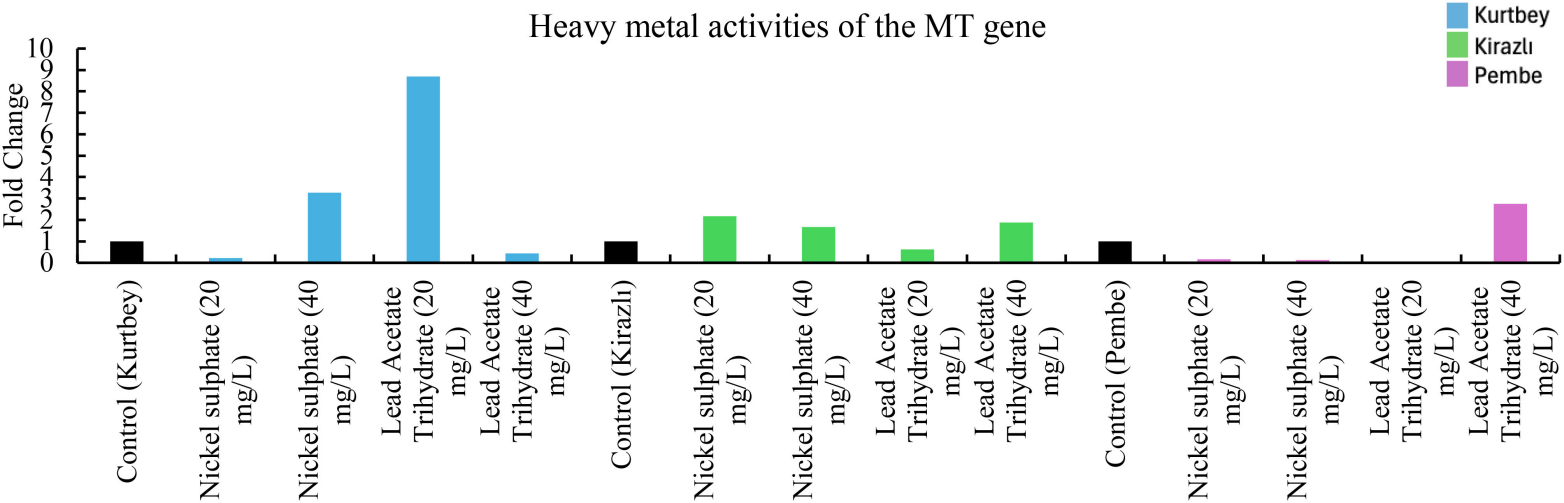
qRT-PCR mRNA gene expression levels of the MT heavy metal stress gene in *Pisum sativum* ssp. *arvense* samples (1-15), Control: non-stressed.

**Figure 4:**
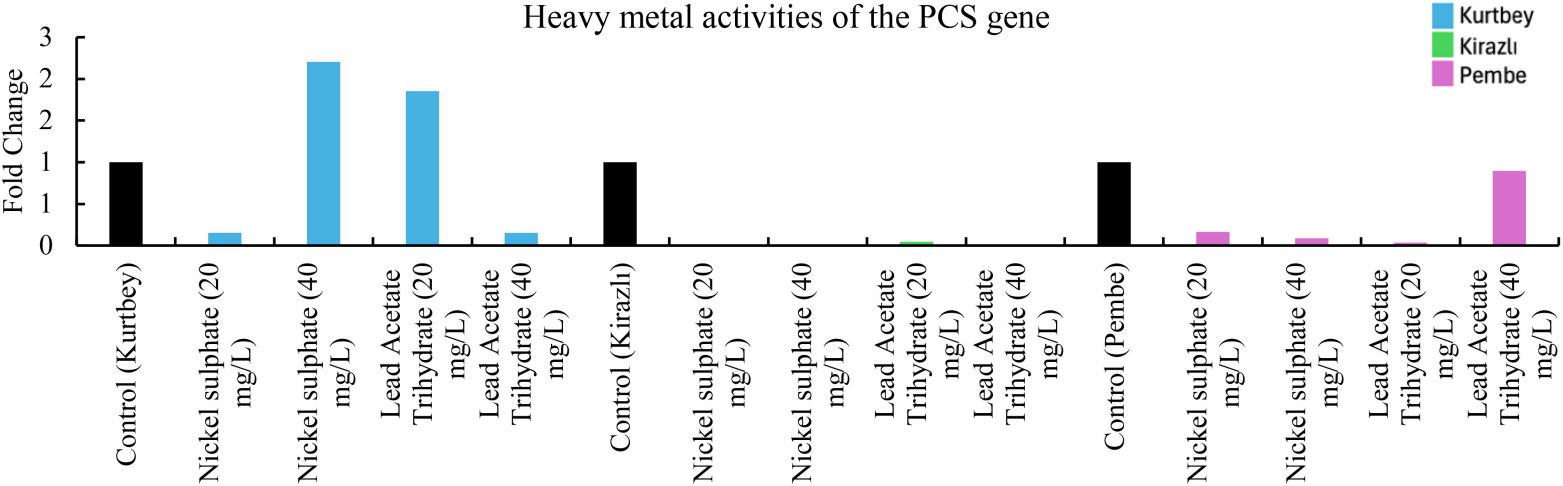
qRT-PCR mRNA gene expression levels of the PCS heavy metal stress gene in *Pisum sativum* ssp. *arvense* samples (1-15), Control: non-stressed.

**Figure 5:**
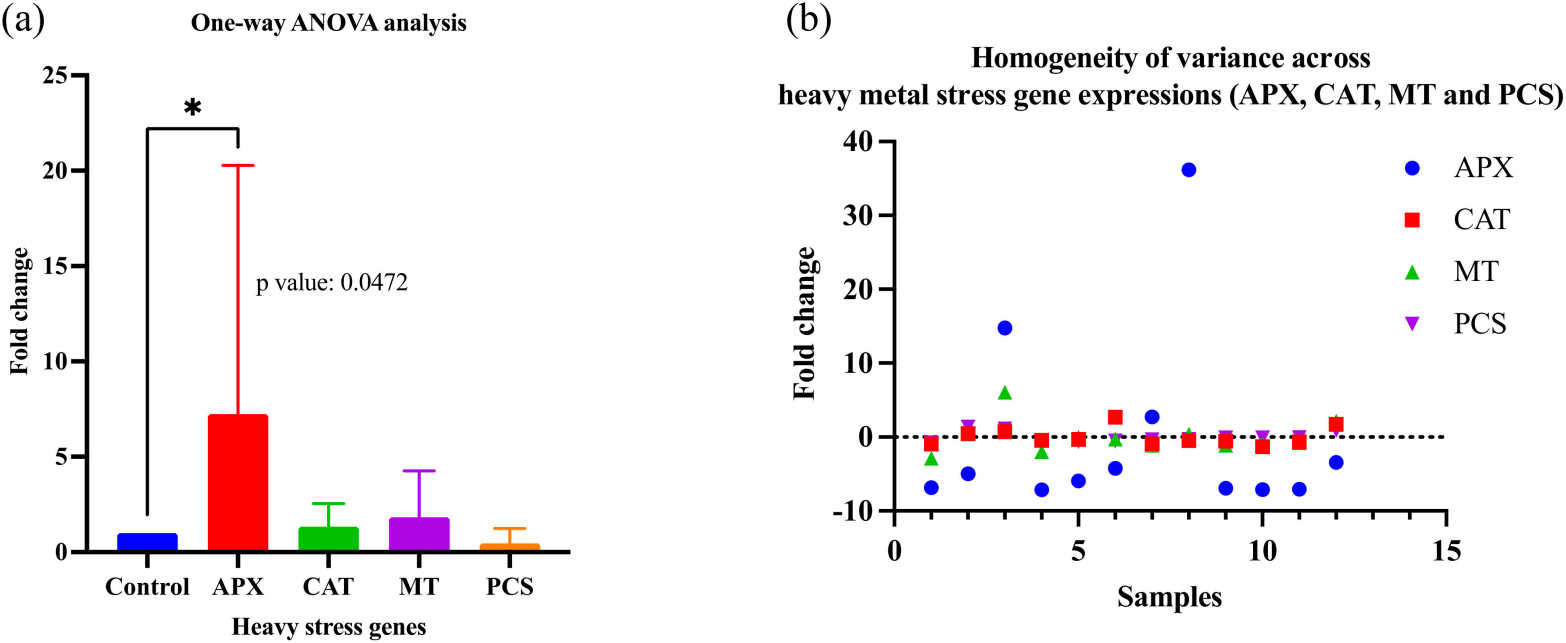
**(a)** One-way ANOVA analysis of heavy metal stress gene expressions (APX, CAT, MT, and PCS) of *Pisum sativum* samples (1-15) compared to control by qRT-PCR (Dunnett’s multiple comparisons test: significant diff. among means, *p<0.05), Control: non-stressed; **(b)** Homogeneity of variance across heavy metal stress gene expressions (APX, CAT, MT and PCS).

In qPCR analysis, the Ct (cycle threshold) value is important in determining the number of cycles that exceed the minimum value (threshold value) required to detect the amount of fluorescent signal. To determine gene expression, the 2^-ΔΔCT^ value needs to be calculated. Calculations were carried out with the following steps (Livak and Schmittgen, 2001). One-way ANOVA analysis of heavy metal stress gene expressions (APX, CAT, MT, and PCS) of forage pea *(Pisum sativum* ssp. *arvense)* samples (1-15) compared to the control by qRT-PCR (**Figures 1**–**4**) was calculated with the Prism 9 program. Additionally, the samples were analysed for Homogeneity of variance among heavy metal stress gene expressions (APX, CAT, MT and PCS) (**Table 2** and **Figure 5**).

**Table 2:** RNA amounts (ng/μl), 260/280 ratio (nm), Cycle Threshold (Ct) values of heavy metal stress-related qRT-PCR analyses of forage pea (*Pisum sativum ssp. arvense*) samples.

Samples (1-15)	RNA amounts (ng/μl)	260/280 ratio (nm)	β-Actin (Ct)	APX (Ct)	CAT (Ct)	MT (Ct)	PCS (Ct)
Control (Kurtbey)	400.41	2.12	30.48	38.83	42.64	31.25	32.84
Nickel sulphate (20 mg/L)	523.86	2.14	30.54	37.59	39.98	29.13	30.19
Nickel sulphate (40 mg/L)	1208.86	2.16	29.59	39.12	42.43	32.07	33.09
Lead Acetate Trihydrate (20 mg/L)	965.808	2.14	29.52	42.33	42.6	33.41	32.77
Lead Acetate Trihydrate (40 mg/L)	1107.76	2.19	29.48	34.8	41.25	29.06	29.12
Control (Kirazlı)	1013.62	2.18	29.46	37.33	42.01	30.89	39.48
Nickel sulphate (20 mg/L)	244.76	2.09	29.53	37.78	41.99	32.08	31.64
Nickel sulphate (40 mg/L)	843.67	2.11	29.55	39.02	44.1	31.72	31.17
Lead Acetate Trihydrate (20 mg/L)	1063.40	2.14	29.48	40.67	40.88	30.23	35.05
Lead Acetate Trihydrate (40 mg/L)	303.69	2.11	28.95	42.26	41.36	31.29	31.65
Control (Pembe İncisi)	1085.23	2.17	27.89	37.19	41.46	30.74	31.3
Nickel sulphate (20 mg/L)	949.83	2.13	29.63	37.18	43.01	29.86	30.43
Nickel sulphate (40 mg/L)	380.809	2.08	29.69	35.87	40.72	29.59	29.57
Lead Acetate Trihydrate (20 mg/L)	368.26	2.10	29.81	36.34	43.22	28.49	28.37
Lead Acetate Trihydrate (40 mg/L)	476.45	2.10	28.83	40.06	44.13	33.14	32.08

## 3 Results and discussion

### 3.1 Plant material and design in forage pea (*Pisum sativum* ssp. *arvense*)

The morphological measurements of forage pea cultivars under varying heavy metal treatments are presented in **Table 3**. Significant differences were observed among cultivars and treatments for all measured traits, including plant height, stem diameter, node number, internode length, stipule width, and stipule length (P < 0.01).

**Table 3:** Morphological measurement results of forage pea.

SOURCES OF VARIATION	Plant height (cm)	Stem diameter (mm)	Node number (nodes/plant)	Internode length (mm)	Stipule width (mm)	Stipule length (mm)
Cultivar	**	**	*	**	ns	**
Treatment	*	ns	ns	ns	ns	*
Variety * Treatment	ns	ns	ns	*	ns	ns
CULTIVAR	Plant height (cm)**	Stem diameter (mm)**	Node number (nodes/plant)*	Internode length (mm)**	Stipule width (mm)	Stipule length (mm)**
Kirazlı	38.86 b	1.27 c	11.80 b	26.34 c	10.85	19.83 b
Kurtbey	48.40 a	1.41 b	13.13 a	31.55 b	12.69	24.18 a
Pembe İncisi	44.80 a	1.64 a	11.40 b	36.98 a	12.10	24.65 a
TREATMENT	Plant height (cm)*	Stem diameter (mm)	Node Number (nodes/plant)	Internode length (mm)	Stipule width (mm)	Stipule length (mm) *
Control	46.88 ab	1.60	11.88	31.27	11.96	22.49 ab
Lead Acetate Triydrate (20 mg/L)	39.66 b	1.37	12.00	30.18	11.36	21.11 b
Lead Acetate Triydrate (40 mg/L)	42.11 ab	1.40	12.22	30.71	11.89	23.15 ab
Nickel Sulphate (20 mg/L)	47.33 a	1.45	12.33	32.05	11.73	22.82 ab
Nickel Sulphate (40 mg/L)	44.11 ab	1.39	12.11	33.91	12.47	24.85 a
CULTIVAR * TREATMENT	Plant height (cm)	Stem diameter (mm)	Node Number (nodes/plant)	Internode length (mm) *	Stipule width (mm)	Stipule length (mm)
Kirazlı * Control	44.00	1.25	11.33	27.41 bc	10.81	19.74
Kirazlı * Lead Acetate Triydrate (20 mg/L)	32.33	1.10	12.66	22.54 c	10.16	17.00
Kirazlı * Lead Acetate Triydrate (40 mg/L)	38.00	1.32	11.00	26.01 bc	11.07	21.84
Kirazlı * Nickel Sulphate (20 mg/L)	43.66	1.41	12.66	30.87 abc	10.90	21.24
Kirazlı * Nickel Sulphate (40 mg/L)	36.33	1.27	11.33	24.88 bc	11.33	19.32
Kurtbey * Control	55.00	1.68	12.66	35.61 abc	12.76	22.66
Kurtbey * Lead Acetate Triydrate (20 mg/L)	43.66	1.39	12.33	30.13 bc	12.60	22.35
Kurtbey * Lead Acetate Triydrate (40 mg/L)	47.33	1.42	14.33	28.13 bc	12.21	24.61
Kurtbey * Nickel Sulphate (20 mg/L)	49.66	1.38	12.66	31.41 abc	12.53	23.65
Kurtbey * Nickel Sulphate (40 mg/L)	46.33	1.20	13.66	32.48 abc	13.37	27.61
Pembe * Control	41.66	1.86	11.66	30.79 abc	12.31	25.07
Pembe * Lead Acetate Triydrate (20 mg/L)	43.00	1.62	11.00	37.88 ab	11.33	23.97
Pembe * Lead Acetate Triydrate (40 mg/L)	41.00	1.47	11.33	37.99 ab	12.39	22.99
PEMBE * Nickel Sulphate (20 mg/L)	48.66	1.56	11.66	33.87 abc	11.75	23.59
Pembe * Nickel Sulphate (40 mg/L)	49.66	1.70	11.33	44.38 a	12.71	27.63
Mean	44.02	1.45	12.11	31.63	11.89	22.89

**P<0.01; *P<0.05; ns: not significant. Lowercase letters within columns indicate statistically significant differences between means based on Tukey’s HSD tests. Bold values represent the overall means for each source of variation. The terms “Cultivar,” “Treatment,” and “Cultivar × Treatment” denote the main effects and interaction effect in the analysis of variance, respectively.

Among the cultivars, Kurtbey consistently exhibited superior performance across multiple morphological traits. Notably, Kurtbey displayed the highest plant height (48.40 cm), stem diameter (1.41 mm), and internode length (31.55 mm). These results suggest that Kurtbey possesses inherent genetic characteristics that confer enhanced growth and development, even under stress conditions. The superior performance of Kurtbey can be attributed to its genetic makeup, which may include genes associated with efficient nutrient uptake, robust cell division, and enhanced stress tolerance. These genetic factors likely contribute to its ability to maintain growth and structural integrity in the presence of heavy metals.

In contrast, Kirazlı exhibited the lowest values for plant height (38.86 cm) and internode length (26.34 mm), indicating a lower tolerance to the applied treatments. Pembe incisi showed intermediate performance, with values between Kurtbey and Kirazlı for most traits.

The heavy metal treatments also significantly affected the morphological traits. Lead acetate trihydrate, particularly at 20 mg/L, resulted in the most pronounced inhibitory effects, leading to reduced plant height and internode length. However, some treatments, such as nickel sulphate at 40 mg/L, increased certain traits, highlighting the complex interactions between heavy metal stress and plant growth.

The significant interaction between cultivar and treatment (P < 0.01) indicates that the response to heavy metal stress varied among cultivars. This suggests that the genetic makeup of each cultivar plays a crucial role in determining its tolerance and adaptation to heavy metal stress. The superior performance of Kurtbey, even under stress conditions, underscores the importance of genetic factors in developing heavy metal-tolerant forage pea cultivars.

**Table 4** presents the correlation coefficients and their corresponding significance levels among various morphological traits in forage pea. The results indicate a strong positive correlation between plant height and internode length (r = 0.5138, p < 0.001), suggesting that taller plants tend to have longer internodes. Similarly, plant height showed a moderate positive correlation with stem diameter (r = 0.3303, p < 0.05) and stipule length (r = 0.5090, p < 0.001). These findings suggest that plant height is influenced by multiple morphological factors.

**Table 4:** Correlation and significance levels of examined traits in fodder pea.

Correlations Probability	Plant height	Stem diameter	Node number	Internode length	Stipule width	Stipule length
**Plant height**	**1.0000**	0.3303	0.2216	0.5138	0.3869	0.5090
	**<.0001**	0.0267*	0.1434	0.0003**	0.0087**	0.0004**
**Stem diameter**	0.3303	**1.0000**	-0.0564	0.4951	0.2083	0.4375
	0.0267*	**<.0001**	0.7126	0.0005**	0.1698	0.0027**
**Node number**	0.2216	-0.0564	**1.0000**	-0.2183	0.0194	0.0301
	0.1434	0.7126	**<.0001**	0.1497	0.8993	0.8444
**Internode length**	0.5138	0.4951	-0.2183	**1.0000**	0.4385	0.6257
	0.0003**	0.0005**	0.1497	**<.0001**	0.0026**	<.0001**
**Stipule width**	0.3869	0.2083	0.0194	0.4385	**1.0000**	0.7304
	0.0087**	0.1698	0.8993	0.0026**	**<.0001**	<.0001**
**Stipule length**	0.5090	0.4375	0.0301	0.6257	0.7304	**1.0000**
	0.0004**	0.0027**	0.8444	<.0001**	<.0001**	**<.0001**

**P<0.01, *P<0.05.

Bold values indicate statistically significant correlations (p < 0.05).

Interestingly, the number of nodes exhibited a negative correlation with internode length (r = -0.2183, p = 0.1497), indicating that plants with more nodes tended to have shorter internodes. This inverse relationship might be attributed to the plant’s allocation of resources between stem elongation and nodal development.

Furthermore, the analysis revealed strong positive correlations among stipule width, stipule length, and internode length. This suggests that these traits are closely linked and may be influenced by similar genetic or environmental factors.

Overall, the correlation analysis highlights the complex relationships among various morphological traits in forage pea. These findings provide valuable insights into the underlying genetic and physiological mechanisms that govern plant growth and development. Understanding these relationships can aid in the development of more efficient breeding strategies for improving forage pea yield and quality.

### 3.2 qRT-PCR analysis of heavy metal stress activities in forage pea (*Pisum sativum* ssp. *arvense*)

As heavy metal contamination continues to pose a significant threat to both the environment and human health, advancing our understanding of plant responses to heavy metal stress is essential for developing effective strategies to mitigate these risks and promote sustainable agricultural practices.

mRNA expression levels of heavy metal stress activities (APX, CAT, MT, and PCS) in forage pea *(Pisum sativum* ssp. *arvense)* samples were investigated by qRT-PCR method. β-Actin was used as a housekeeping control gene. Nickel sulphate (20 mg/L and 40 mg/L) and lead acetate trihydrate (20 mg/L and 40 mg/L) treated samples were compared with an untreated plant sample as a negative control.

When APX mRNA expressions in heavy metal stress activity were compared to the control (Kurtbey), in nickel sulphate (20 mg/L) and lead acetate trihydrate (40 mg/L) applications, expression was suppressed, and tolerance was detected. However, in nickel sulphate (40 mg/L) and lead acetate trihydrate (20 mg/L) applications, an increase in expression (2- and 22-fold change, respectively) was determined. According to APX mRNA expressions, heavy metal amount and tolerance rate are inversely proportional in nickel sulphate and lead acetate trihydrate applications. No increase in gene expression was detected in high dose (40 mg/L) application of lead acetate trihydrate.

When APX mRNA expressions were compared with the control (Kirazlı), an increase in expression (0.3-, 3-,10- and 43-fold change, respectively) was determined in nickel sulphate (20-40 mg/L) and lead acetate trihydrate (20-40 mg/L) applications. Lead acetate trihydrate has a higher heavy metal stress rate than nickel sulphate. This stress rate is directly proportional to the amount of heavy metal application.

When APX mRNA expressions were compared with the control (Pembe İncisi), expression was suppressed, and tolerance was detected in nickel sulphate (20-40 mg/L) and lead acetate trihydrate (20 mg/L) applications. An increase in expression (approximately 2-fold change) was detected only in lead acetate trihydrate (40 mg/L) (**Figure 1**).

When CAT mRNA expressions in heavy metal stress activity were compared to the control (Kurtbey), in nickel sulphate (20 mg/L) and lead acetate trihydrate (40 mg/L) applications, expression was suppressed, and tolerance was detected. However, in nickel sulphate (40 mg/L) and lead acetate trihydrate (20 mg/L) applications, an increase in expression (1.6- and 1.9-fold change, respectively) was determined. According to CAT mRNA expressions, heavy metal amount and tolerance rate are inversely proportional in nickel sulphate and lead acetate trihydrate applications. No increase in gene expression was detected in high dose (40 mg/L) application of lead acetate trihydrate.

When CAT mRNA expressions were compared with the control (Kirazlı), an increase in expression (4-fold change) was determined only in nickel sulphate (40 mg/L). CAT mRNA expressions were suppressed and tolerated in other chemical applications.

When CAT mRNA expressions were compared with the control (Pembe İncisi), an increase in expression (3.3-fold change) was determined only in lead acetate trihydrate (40 mg/L). CAT mRNA expressions were suppressed and tolerated in other chemical applications (**Figure 2**).

When MT mRNA expressions in heavy metal stress activity were compared to the control (Kurtbey), in nickel sulphate (20 mg/L) and lead acetate trihydrate (40 mg/L) applications, expression was suppressed, and tolerance was detected. However, in nickel sulphate (40 mg/L) and lead acetate trihydrate (20 mg/L) applications, an increase in expression (3.3- and 8.7-fold change, respectively) was determined. According to MT mRNA expressions, heavy metal amount and tolerance rate are inversely proportional in nickel sulphate and lead acetate trihydrate applications. No increase in gene expression was detected in high dose (40 mg/L) application of lead acetate trihydrate.

When MT mRNA expressions in heavy metal stress activity were compared to the control (Kirazlı), in lead acetate trihydrate (20 mg/L), expression were suppressed and tolerated. However, in nickel sulphate (20-40 mg/L) and lead acetate trihydrate (40 mg/L) applications, an increase in expression (2.1-, 1.6- and 1.9-fold change, respectively) was determined.

When MT mRNA expressions were compared with the control (Pembe İncisi), an increase in expression (2.8-fold change) was determined only in lead acetate trihydrate (40 mg/L). MT mRNA expressions were suppressed and tolerated in other chemical applications. (**Figure 3**).

When PCS mRNA expressions in heavy metal stress activity were compared to the control (Kurtbey), in nickel sulphate (20 mg/L) and lead acetate trihydrate (40 mg/L) applications, expression was suppressed, and tolerance was detected. However, in nickel sulphate (40 mg/L) and lead acetate trihydrate (20 mg/L) applications, an increase in expression (3.3- and 8.7-fold change, respectively) was determined. According to PCS mRNA expressions, heavy metal amount and tolerance rate are inversely proportional in nickel sulphate and lead acetate trihydrate applications. No increase in gene expression was detected in high dose (40 mg/L) application of lead acetate trihydrate.

When PCS mRNA expressions in heavy metal stress activity were compared with the control (Kirazlı and Pembe İncisi), expression was suppressed and tolerated in all chemical applications as nickel sulphate (20-40 mg/L) and lead acetate trihydrate (20-40 mg/L). PCS mRNA heavy metal stress treatments (Kirazlı and Pembe İncisi) showed similar results (**Figure 4**).

Significant differences were observed in both morphological measurements and molecular level analyses of forage pea varieties under varying heavy metal treatments. Among the varieties, Kurtbey consistently showed superior performance in multiple morphological traits. It shows that Kurtbey has natural genetic traits that provide enhanced growth and development even under stress conditions. When APX, CAT, MT and PCS mRNA expressions in heavy metal stress activity were compared with the control (Kurtbey), expression was suppressed, and stress was tolerated in nickel sulfate (20-40 mg/L) and lead acetate trihydrate (40 mg/L) treatments. Heavy metal stress did not affect morphological trait performance in Kurtbey. This may indicate the effect of strong genetic factors.

In contrast, Kirazlı exhibited the lowest values ​​in morphological traits and showed lower tolerance to heavy metal applications. However, when heavy metal stress activity, especially PCS mRNA expressions, were compared with the control (Kirazlı), expression was suppressed in nickel sulfate (20-40 mg/L) and lead acetate trihydrate (20-40 mg/L) applications and stress was tolerated. The effect of heavy metal stress on morphological trait performance in Kirazlı was high. This situation can be explained by the fact that genetically strong tolerance to heavy metal stress caused weak morphological traits in the plant. Pembe İncisi showed an intermediate performance between Kurtbey and Kirazlı in most morphological traits. When heavy metal stress activity, APX, CAT, MT and PCS mRNA expressions were compared with the control (Pembe İncisi), expression was suppressed in applications other than lead acetate trihydrate (40 mg/L) and stress was tolerated. Pembe Pearl has more effective stress tolerance performance at the gene expression level compared to Kurtbey and Kirazlı. This situation can be interpreted as the effect of medium-level performance in morphological traits with high stress tolerance performance at the gene level.

Research has shown that heavy metal exposure induces oxidative stress in plants, leading to the generation of reactive oxygen species (ROS). The expression of antioxidant genes such as APX and CAT is significantly upregulated in response to heavy metals like cadmium and lead. For instance, Małecka et al. reported that lead stress in pea root cells resulted in increased ROS generation, which subsequently activated the antioxidative defence mechanisms, including the upregulation of APX and CAT genes (Małecka et al., 2001). Similarly, El-Amier et al. demonstrated that heavy metal stress enhances the activity of superoxide dismutase (SOD), which is closely linked to the expression of APX genes, indicating a coordinated response to oxidative stress (El-Amier et al., 2019).

Rahman et al., 2017 study investigated the effect of silicon (Si) on Cd toxicity in forage pea at biochemical and molecular levels. In plants under Cd stress, GSH (phytochelatin precursor) and MT (metallothionein) transcripts were expressed in roots. In addition, CAT, POD, SOD and GR activity reduced Cd toxicity via silicon (Rahman et al., 2017).

Metallothioneins (MTs) and phytochelatins (PCS) are critical for heavy metal detoxification in plants. They bind to heavy metals, facilitating their sequestration and reducing their toxicity. In a study by Belimov et al., the cadmium-tolerant pea mutant SGECdt exhibited increased expression of MT genes, which contributed to enhanced cadmium accumulation and tolerance (Belimov et al., 2018). Furthermore, Tsyganov et al. highlighted the role of MTs in the detoxification of heavy metals, suggesting that these proteins are essential for maintaining cellular homeostasis under metal stress. The PCS gene, responsible for phytochelatin synthesis, also plays a pivotal role in heavy metal detoxification. Research indicates that PCS expression is upregulated in response to cadmium exposure, facilitating the synthesis of phytochelatins that bind cadmium ions and mitigate their toxic effects (Tsyganov et al., 2020).

According to Eid et al., 2020; heavy metal bioaccumulation, growth, and yield of *Pisum sativum* ssp. *arvense* (forage pea) grown in agricultural soil amended with sewage sludge (SS). Heavy metals such as Co, Cu, Mn, Ni, Zn, Cd, Cr, and Pb in soil samples increased with SS application rates. All heavy metals except Cd in the shoot of *P. sativum* increased significantly (Eid et al., 2020).

Galal et al., 2021 study examined the heavy metals cadmium (Cd), arsenic (As), chromium (Cr), copper (Cu), nickel (Ni), iron (Fe), manganese (Mn), zinc (Zn), silver (Ag), cobalt (Co) and vanadium (V) in *Pisum sativum*. It was shown that the bioaccumulation factor exceeded by Pb, Cd, Fe and Mn poses potential health risks in food crops or animal feeds that accumulate heavy metals (Galal et al., 2021).

El-Okkiah et al., 2022 study investigated the Cd toxicity of *Pisum sativum* plant. Cd accumulation in pea tissue reduced plant growth, leaf area, and shoot and root dry weights. In addition, lower chlorophyll levels were seen in plants due to the effect of Cd. In addition, oxidative damage was detected according to hydrogen peroxide (H_2_O_2_) and malondialdehyde (MDA) levels (El-Okkiah et al., 2022).

Górski and Płaza, 2023, determined the effect of a mixture of forage pea (*Pisum sativum*) and spring triticale (*Triticosecale* Wittm.) on the content of heavy metals (Cu, Zn, Cd, Pb, Cr and Ni). According to the data, the content of cadmium and lead in the mixture of forage pea and spring triticale was below the detection threshold (Górski and Płaza, 2023).

Wysokinski et al., 2023, examined the changes in the content, uptake rate, accumulation and transport of heavy metals in the development of pea plants. The content of heavy metals in the plant, bioaccumulation factors (BAFs) and translocation factors (TFs) were determined. According to BAF, excessive accumulation of lead and zinc in pea plants indicated the potential for moderate accumulation of other heavy metals. It was explained that peas should not be used as human food and animal feed due to excessive lead concentration (Wysokinski et al., 2023).

According to Saleem et al., 2024; The effect of Cadmium citrate on Zn-Lys application in *Pisum sativum* plant was investigated. Cd toxicity decreased plant growth, chlorophyll content, osmoprotectants and anthocyanin content, while an increase in MDA, H_2_O_2_ initiation, enzymatic antioxidant activities and phenolic, flavonoid, proline was observed. After Zn-Lys application in the plant, plant growth, biomass, photosynthetic qualities, osmoprotectants and anthocyanin content increased, while a further increase in enzymatic antioxidant activity, total phenolic, flavonoid and proline contents were detected. However, a decrease in MDA and H_2_O_2_ levels was determined (Saleem et al., 2024).

Naveed et al, in their 2024 study, indicated that sewage water is widely used for irrigation to increase agricultural productivity and evaluated the effects of this water on heavy metals, compounds in soil and pea plants. Pea plants were irrigated with tap water (TW), sewage water (SW) and tap + sewage water (TW + SW). Biochar and polyacrylamide (PAM) were mixed into the soil. Cd and Cr concentration in biochar, PAM, SW and TW + SW applied soil decreased. Irrigation with TW + SW, application of PAM and biochar remediated the contaminated soil and proposed an effective strategy for plant growth and yield in *Pisum sativum* (Naveed et al., 2024).

In our study, in the application of 40 mg/L nickel sulphate, APX (0.6-fold change, 60%, Pembe İncisi), CAT (0.8-fold change, 80%, Pembe), MT (0.9-fold change, 90%, Pembe İncisi), PCS (0.9-fold change, 90%, Kirazlı and Pembe İncisi) mRNA expressions were down-regulated and heavy metal stress was tolerated. In addition, at 40 mg/L of Lead Acetate Trihydrate application, APX (0.9-fold change, 90%, Kurtbey), CAT (0.2-fold change, 20%, Kurtbey and 0.1-fold change, 10%, Kirazlı), MT (0.6-fold change, 60%, Kirazlı), PCS (0.8-fold change, 80%, Kurtbey, 0.9-fold change, 90%, Kirazlı and 0.1-fold change, 10%, Pembe) mRNA expressions were down-regulated and heavy metal stress was tolerated.

In 20 mg/L nickel sulphate and 40 mg/L Lead Acetate Trihydrate applications, APX, CAT, MT, and PCS gene expressions were down-regulated in Kurtbey and heavy metal stress was tolerated. Moreover, APX, CAT, MT, and PCS gene expressions were down-regulated in Pembe in 20-40 mg/L nickel sulphate and 20 mg/L Lead Acetate Trihydrate applications and heavy metal stress was tolerated.

Remarkably, the one-way ANOVA analysis (Dunnett’s multiple comparisons test: diff. among means) of the heavy metal stress gene expressions (APX, CAT, MT, and PCS) of *Pisum sativum* samples (1-15) compared to the control was found to be significant (APX, p value: 0.0472). One-way ANOVA analysis of *Pisum sativum* samples was evaluated by comparing cultivars (Kurtbey, Kirazlı and Pembe İncisi) with heavy metal stress-related gene expressions and control. According to Dunnett’s multiple comparisons test, the difference between the means (p>0.05) is not significant.

There may be several main reasons for the different stress responses of various cultivars of Pisum species exposed to the same heavy metal stress genes. Among the cultivars, Kurtbey consistently showed superior performance in multiple morphological traits. Heavy metal stress did not affect the morphological trait performance in Kurtbey. Kurtbey has more resistant genetic traits in the capacity to cope with stress. It may have developed effective adaptation strategies against environmental conditions and stresses. It has an efficient antioxidant defence system in neutralizing free radicals and preventing cell damage. Kirazlı showed the lowest values ​​in morphological traits and showed lower tolerance to heavy metal applications except for the PCS gene. Kirazlı may not have resistant genetic traits, a strong antioxidant defence system in neutralizing free radicals and preventing cell damage. It may also be inadequate in developing effective adaptation against environmental conditions and stresses. In addition, its physiological traits (water intake, nutrient transport, toxic substance excretion) may have affected heavy metal accumulation, toxic effects and stress responses. Pembe İncisi has a medium level of performance in morphological characteristics, while it has a high stress tolerance performance in heavy metal stress gene activity level. These results add awareness to the protection, production, cultivation of plant cultures or the potential to obtain the targeted yield.

## 4 Conclusion

This study aimed to assess the impact of nickel sulfate and lead acetate trihydrate on the morphological characteristics of three forage pea cultivars (Kurtbey, Pembe incisi, and Kirazlı) in a greenhouse experiment conducted in Pazar district, Rize, Türkiye, in 2024. Plants were exposed to different concentrations of heavy metals (0, 20, and 40 mg/L) for four weeks after sowing. Morphological parameters, including plant height, stem diameter, node number, internode length, stipule width, and stipule length, were measured and analyzed using ANOVA and Tukey’s HSD test.

The study of heavy metal genes such as APX, CAT, MT, and PCS in forage pea (*Pisum sativum* ssp. *arvense*) has garnered significant attention due to the plant’s sensitivity to heavy metal stress and its potential for phytoremediation. These genes play crucial roles in the plant’s antioxidant defence mechanisms and metal detoxification processes, which are vital for survival in contaminated environments.

By applying nickel sulphate and lead acetate trihydrate compounds to forage pea *(Pisum sativum* ssp. *arvense)* species, APX, CAT, MT and PCS heavy metal stress activities were analysed by qPCR method.

Due to the stress factor, nickel sulphate (20 mg/L) application is associated with a decrease in APX, CAT and PCS mRNA expressions and nickel sulphate (40 mg/L) application is associated with an increase in APX, CAT, MT and PCS mRNA expressions. Due to the stress factor, lead acetate trihydrate (20 mg/L) application is associated with a decrease in APX (only Pembe), CAT (Kirazlı and Pembe), MT (Kirazlı and Pembe İncisi) and PCS mRNA expressions and lead acetate trihydrate (40 mg/L) application is associated with an increase in APX (Kirazlı and Pembe İncisi), CAT (Kirazlı and Pembe İncisi), MT (Kirazlı and Pembe İncisi) and PCS mRNA expressions.

According to the results of one-way ANOVA analysis (Dunnett’s multiple comparisons test: diff. among means), significance was determined in APX gene expressions due to heavy metal stress in *Pisum sativum* ssp. *arvense* samples compared to the control by qRT-PCR. Health risks associated with heavy metal accumulation in plants are a critical area of ​​research. Heavy metals can enter the food chain through contaminated crops and pose significant health risks to humans and animals. Therefore, our study investigating heavy metal stress in plants will not only help develop remediation strategies but also inform food safety regulations and guidelines to protect public health.

## Data Availability

The original contributions presented in the study are included in the article. Further inquiries can be directed to the corresponding author.

## References

[B1] Acar Z. Ayan I. (2012). Culture of forage crops (Türkiye: Ondokuz Mayis University, Agriculture Faculty). Textbook No:2.

[B2] Acikgoz E. (2001). Forage crops (Türkiye: Uludag University, Agriculture Faculty, Department of Field Crops, Uludag University Empowerment Foundation). No:182.

[B3] Acikgoz E. (2013). Forage crops breeding (Türkiye: Dairy Livestock Training Center Publications). No:8, s.41.

[B4] Avcioglu R. Hatipoglu R. Karadag Y. (2009). Leguminous forage crops volume II (Türkiye: Ministry of Food, Agriculture and Livestock, Publications of the General Directorate of Agriculture and Publication).

[B5] Belimov A. A. Malkov N. V. Puhalsky J. V. Tsyganov V. E. Bodyagina K. B. Safronova V. I.. (2018). The crucial role of roots in increased cadmium-tolerance and cd-accumulation in the pea mutant *SGECd^t^ * . Biol. Plantarum 62, 543–550. doi: 10.1007/s10535-018-0789-0

[B6] Eid E. M. El-Bebany A. F. Taher M. A. Alrumman S. A. Galal T. M. Shaltout K. H.. (2020). Heavy metal bioaccumulation, growth characteristics, and yield of Pisum sativum L. grown in agricultural soil-sewage sludge mixtures. Plants 9, 1300. doi: 10.3390/plants9101300 33019617 PMC7601226

[B7] El-Amier Y. Elhindi K. El-Hendawy S. Al-Rashed S. Abd-ElGawad A. (2019). Antioxidant system and biomolecules alteration in pisum sativum under heavy metal stress and possible alleviation by 5-aminolevulinic acid. Molecules 24, 4194. doi: 10.3390/molecules24224194 31752309 PMC6891517

[B8] El-Okkiah S. A. El-Tahan A. M. Ibrahim O. M. Taha M. A. Korany S. M. Alsherif E. A.. (2022). Under cadmium stress, silicon has a defensive effect on the morphology, physiology, and anatomy of pea (Pisum sativum L.) plants. Front. Plant Sci. 13, 997475. doi: 10.3389/fpls.2022.997475 36325574 PMC9621089

[B9] Galal T. M. Hassan L. M. Ahmed D. A. Alamri S. A. Alrumman S. A. Eid E. M. (2021). Heavy metals uptake by the global economic crop (Pisum sativum L.) grown in contaminated soils and its associated health risks. PloS One 16, e0252229. doi: 10.1371/journal.pone.0252229 34086714 PMC8177654

[B10] Górski R. Płaza A. (2023). Heavy metal content in green matter of field pea and spring triticale mixtures and their usage determination as green fodder for animals. Zemdirbyste-Agriculture 110, 27–32. doi: 10.13080/z-a.2023.110.004

[B11] Jamil M. Malook I. Rehman S. U. Aslam M. M. Fayyaz M. Shah G.. (2024). Inoculation of heavy metal resistant bacteria alleviated heavy metal-induced oxidative stress biomarkers in spinach (spinacia oleracea l.). BMC Plant Biol. 24, 1–14. doi: 10.1186/s12870-024-04757-7 PMC10976752 38539080

[B12] Livak K. J. Schmittgen T. D. (2001). Analysis of relative gene expression data using real-time quantitative PCR and the 2^– ΔΔCT^ method. methods 25, 402–408. doi: 10.1006/meth.2001.1262 11846609

[B13] Małecka A. Jarmuszkiewicz W. Tomaszewska B. (2001). Antioxidative defense to lead stress in subcellular compartments of pea root cells. Acta Biochim. Polonica 48, 687–698. doi: 10.18388/abp.2001_3903 11833777

[B14] Naveed M. Fatima M. Naseem Z. Ahmad Z. Gaafar A. R. Z. Shabbir M.. (2024). Improving the growth of pea plant by biochar–polyacrylamide association to cope with heavy metal stress under sewage water application in a greenhouse. Front. Environ. Sci. 12, 1380867. doi: 10.3389/fenvs.2024.1380867

[B15] Priya A. Muruganandam M. Ali S. S. Kornaros M. (2023). Clean-up of heavy metals from contaminated soil by phytoremediation: a multidisciplinary and eco-friendly approach. Toxics 11, 422. doi: 10.3390/toxics11050422 37235237 PMC10221411

[B16] Rahman M. F. Ghosal A. Alam M. F. Kabir A. H. (2017). Remediation of cadmium toxicity in field peas (Pisum sativum L.) through exogenous silicon. Ecotoxicology Environ. Saf. 135, 165–172. doi: 10.1016/j.ecoenv.2016.09.019 27736676

[B17] Saleem M. H. Parveen A. Perveen S. Akhtar N. Abasi F. Ehsan M.. (2024). Alleviation of cadmium toxicity in pea (Pisum sativum L.) through Zn– Lys supplementation and its effects on growth and antioxidant defense. Environ. Sci. Pollution Res. 31, 10594–10608. doi: 10.1007/s11356-024-31874-5 38198090

[B18] Sharma J. K. Kumar N. Singh N. Santal A. R. (2023). Phytoremediation technologies and their mechanism for removal of heavy metal from contaminated soil: an approach for a sustainable environment. Front. Plant Sci. 14. doi: 10.3389/fpls.2023.1076876 PMC9911669 36778693

[B19] Soumya V. Kiranmayi P. Kumar K. (2022). Morpho-anatomical responses of catharanthus roseus due to combined heavy metal stress observed under scanning electron microscope. Plant Sci. Today. 9, 623–631. doi: 10.14719/pst.1621

[B20] Tsyganov V. E. Tsyganova A. V. Gorshkov A. P. Seliverstova E. V. Kim V. E. Chizhevskaya E. P.. (2020). Efficacy of a plant-microbe system: pisum sativum (l.) cadmium-tolerant mutant and rhizobium leguminosarum strains, expressing pea metallothionein genes psmt1 and psmt2, for cadmium phytoremediation. Front. Microbiol. 11. doi: 10.3389/fmicb.2020.00015 PMC7000653 32063892

[B21] Wysokinski A. Kuziemska B. Lozak I. (2023). Heavy metal allocation to pea plant organs (Pisum sativum L.) from soil during different development stages and years. Agronomy 13, 673. doi: 10.3390/agronomy13030673

[B22] Yalçın V. Leblebici Z. (2014). Bazı ağır metallerin (Pb, Cd, Ni) sucul bitkiler (Salvinia natans (L.) All., Lemna minor L.) üzerinde yaptığı stres ve biyolojik yanıtla (Türkiye: Nevşehir Hacı Bektaş Veli Üniversitesi).

